# Origin of the Words Denoting Some of the Most Ancient Old World Pulse Crops and Their Diversity in Modern European Languages

**DOI:** 10.1371/journal.pone.0044512

**Published:** 2012-09-04

**Authors:** Aleksandar Mikić

**Affiliations:** Institute of Field and Vegetable Crops, Novi Sad, Serbia; University of Arkansas, United States of America

## Abstract

This preliminary research was aimed at finding the roots in various Eurasian proto-languages directly related to pulses and giving the words denoting the same in modern European languages. Six Proto-Indo-European roots were indentified, namely *arnk(')-* (‘a leguminous plant’), *bhabh- (‘field bean’), *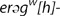 (‘a kernel of leguminous plant’, ‘pea’), *ghArs-* (‘a leguminous plant’), *kek- (‘pea’) and *lent- (‘lentil’). No Proto-Uralic root was attested save hypothetically *kača (‘pea’), while there were two Proto-Altaic roots, *bŭkrV (‘pea’) and *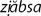 (‘lentil’). The Proto-Caucasianx root *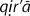 denoted pea, while another one, *hōwł(ā) (‘bean’, ‘lentil’) and the Proto-Basque root *iłha-r (‘pea’, ‘bean’, ‘vetch’) could have a common Proto-Sino-Caucasian ancestor, *hVwłV (‘bean’) within the hypothetic Dené-Caucasian language superfamily. The Modern Maltese preserved the memory of two Proto-Semitic roots, *'adaš- (‘lentil’) and *pūl- (‘field bean’). The presented results prove that the most ancient Eurasian pulse crops were well-known and extensively cultivated by the ancestors of all modern European nations. The attested lexicological continuum witnesses the existence of a millennia-long links between the peoples of Eurasia to their mutual benefit. This research is meant to encourage interdisciplinary concerted actions between plant scientists dealing with crop evolution and biodiversity, archaeobotanists and language historians.

## Introduction

It may be said that the term *pulse* has an identical meaning to *food legume*, with both denoting those grain legumes used exclusively for human consumption, mostly in the form of immature (green) pods, immature (green) grains and mature (dry) grains. Among the most important pulses in temperate regions are pea (*Pisum sativum* L.), field bean (*Vicia faba* L.), lentil (*Lens culinaris* Medik.) and chickpea (*Cicer arietinum* L.). Pulses once significant, but today largely neglected, are the bitter vetch (*Vicia ervilia* (L.) Willd.) and grass pea (*Lathyrus sativus* L.). Many pulses are, in fact, multifunctional crops that may be also used for animal feed, as green forage, forage dry matter (hay), forage meal, silage, haylage or straw (1), or as green manure: a valuable feature in contemporary trends such as organic farming and sustainable agriculture (2). Most traditional Eurasian pulses originated in either the Near Eastern centre of diversity, such as the pea, lentil, chickpea and common vetch (*Vicia sativa* L.); or the Mediterranean, such as the grass pea, red vetchling (*Lathyrus cicera* L.) and bitter vetch; or the Central Asian, such as the field bean (3). As other plant species used for food, pulses were first collected by hunter-gatherers.

**Figure 1:**
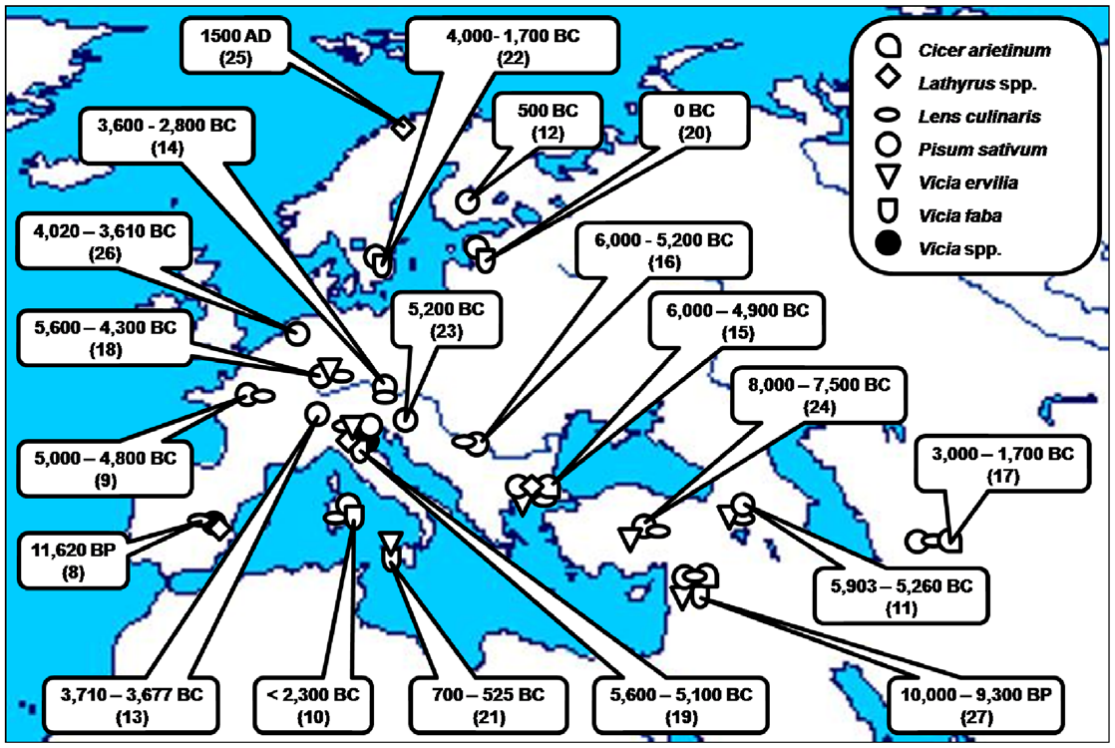
Some of the oldest archaeobotanical evidence related to the first domesticated pulse crops in Europe and its neighbouring regions.

Among the oldest finds of pulses are those of lentil and bitter vetch in Franchthi cave in Greece, dated to about 11,000 BC (4). Pulses are also considered one of the first domesticated plant species, and thus the first crops (5), with much archaeobotanical evidence, mainly from present-day Syria (6). Together with cereals, pulses were part of the ‘agricultural revolution’ in post-glacial Europe (7), quickly spreading over the entire continent (Fig. 1). Pulse seeds are more degradable than those of cereals, and are usually found in smaller amounts, except in a few cases such as at Hissar in south Serbia, where thousands of charred pea and bitter vetch seeds were found, but almost no cereals (28). There has been a growing interest by molecular biologists in extracting ancient DNA (aDNA) from charred and other preserved old seeds, with recent reports on its success in the case of the pea and bitter vetch (29).

The European continent has always been rich in a linguistic sense: it is estimated that it is (or was) home to at least 300 extinct and living languages (30). The most widely-spoken family there is the Indo-European, with all its branches, namely Albanian, Armenian, Baltic, Celtic, Germanic, Hellenic, Indo-Iranian, Italic and Slavic. It’s commonly regarded that the extinct and the living languages of all these branches had a common ancestor, usually referred to as Proto-Indo-European. Although the exact position of the original homeland of the Proto-Indo-Europeans (i.e. the people who actually spoke Proto-Indo-European) still remains uncertain, the widely-accepted Kurgan hypothesis proposes it was the wide Pontic-Caspian steppe, from 4,500 BC to 2,500 BC (31), following which great migrations began in many directions over Europe and Asia, and Proto-Indo-European started to produce numerous derivatives (32).

The Uralic language family descended from Proto-Uralic, most likely spoken on the eastern slopes of the Ural Mountains millennia ago (33). It developed into two branches, Finno-Ugric and Samoyedic, with the former spreading over northernmost Europe. Similarly, the Altaic language family slowly evolved from the Proto-Altaic, developing (most likely) in western regions of Siberia and diversifying into Turkic, Mongolic and a few other branches (34). The Caucasus is home to two more language families, namely Caucasian, also known as North Caucasian, and by some theories linked with the Basque language isolate into a Dené-Caucasian superfamily (35), and Kartvelian, or South Caucasian, languages, with Georgian as the best-known representative. Finally, Maltese remains as the only genuinely European language belonging to the Semitic branch of the great Afro-Asiatic family (36).

Viewing the said archaeobotanical and linguistic evidence the one in the light of the other, it might be assumed that the pulses were surely among the plant species, from both wild and agricultural floras, which were familiar to the ancestors of the modern European nations during their complex ethnic evolution. The mechanisms underlying the genetic, ethnic and linguistic development of each of the great European language families are still far from explained in a detailed and satisfactory way, and the frequent migrations of each, along with numerous mutual cultural contacts, make this issue even harder to comprehend. One must allow the possibility that the spectra of crop usages, and of words denoting such crops, were manifold. On arrival, newcomers could find cultivated the crop they themselves had grown in their old homeland, and either retain their original word or adopt a new one from the aboriginal population. Also, the introduction of a new technology, or a novel way of using an already cultivated crop, by a neighboring people could also introduce words that would replace old ones. In any event, common vocabularies related to diverse aspects of the everyday life of the ancestral members of one language family are still well preserved, albeit to a varying extent, by the Indo-European languages in particular (37). Among common words, those denoting various kinds of food are regularly found in every European language family: and words or names for pulses are ones that should be most prominently represented (38).

This preliminary research had two main goals. The first one was to find those root-words in various protolanguages whose primeval meaning was directly related to pulses and which, in most cases, begot the words denoting the same in modern European languages. It is also a call by plant scientists dealing with crop evolution and biodiversity to archaeobotanists and language historians, to combine their separate efforts in joint and concerted action towards a more complete and better-comprehended discernment of the dawn of pulse crop cultivation in the Old World.

## Results and Discussion

### Indo-European Languages

The Indo-European language family proved to be the richest in root-words originally relating to pulse crops. The meaning of the Proto-Indo-European root *arnk(')-, *arenko-* was literally *a leguminous plant* (39, 40). It was preserved only in Old Greek, where the word *άρακος* also generally denoted a leguminous plant or, specifically, the annual vetchling (*Lathyrus annuus* L.). Its descendant in Modern Greek, *αρακάς*, also denotes the pea. It is noteworthy that this Proto-Indo-European root-word was immortalized in plant taxonomy by Linneaus as *Arachis* L., denoting the groundnut genus (41).

**Figure 2:**
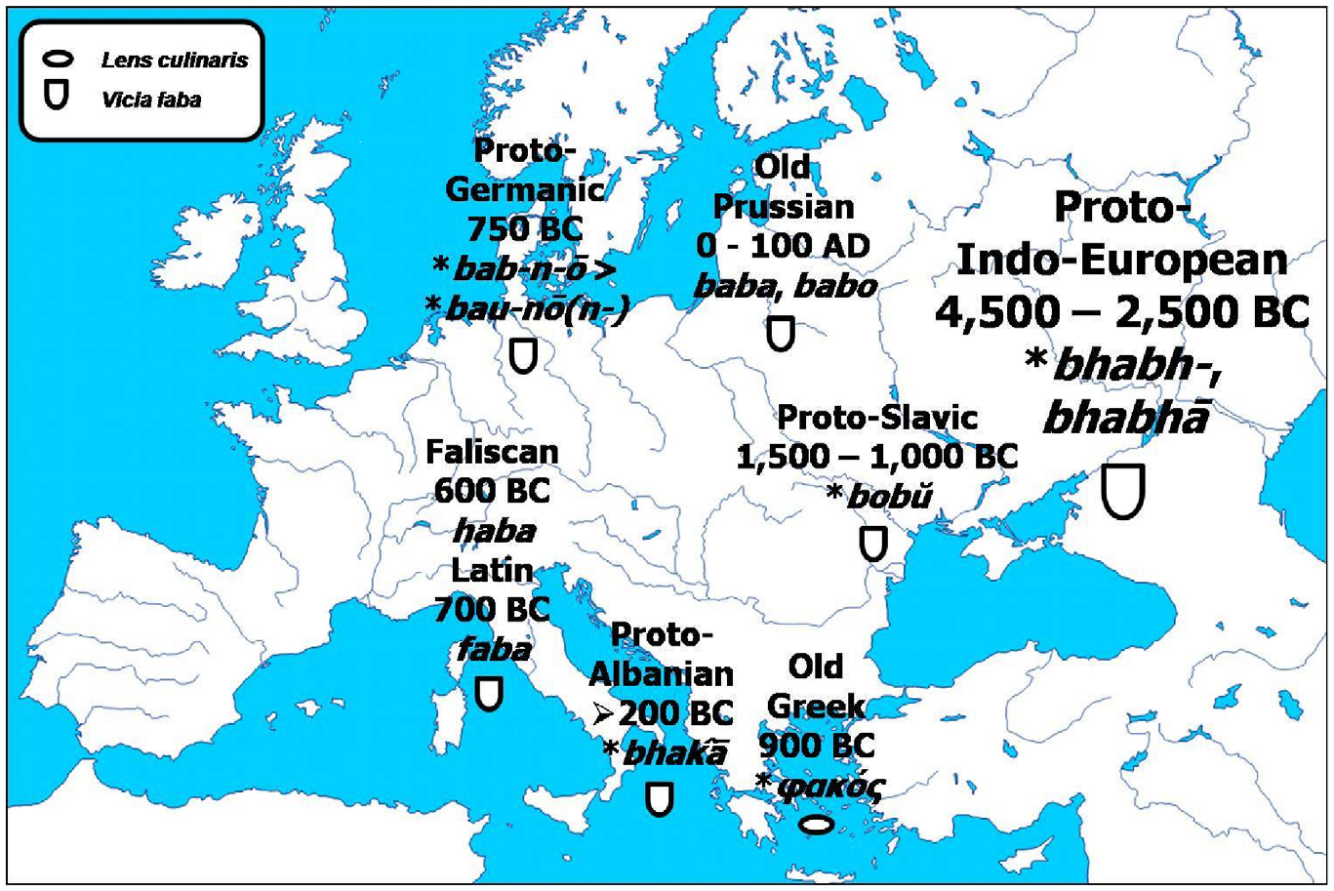
Initial evolution of the Proto-Indo-European root *bhabh-.

One of the Proto-Indo-European roots related to pulses with a large number of attested direct derivatives (Fig. 2) is *bhabh-, *bhabhā*. It is regarded that the literal meaning of this root was a descriptive one, *swollen, swelling*, and was used to denote the field bean (39, 40). Despite the distance of many millennia between this Proto-Indo-European word-root and its countless descendants in the modern Indo-European languages, the original meaning has been fully preserved. For example (Table 1), the Proto-Albanian *bhakā gave the Modern Albanian *bathe*; the unattested Proto-Baltic root-word, probably similar to the Old Prussian *baba*, *babo*, gave the Modern Lithuanian *pupa*; the Proto-Germanic *bau-nō(n-) gave the Modern Danish *bønne*, the Standard German *Bohne* and the English *bean*; the Latin, a descendant of the unattested Proto-Italic together with the extinct Faliscan language, gave the Modern Italian *fava*; the Modern Spanish *haba* and the Modern Sardinian *fa*; the Proto-Slavic *bobŭ gave the Modern Polish *bób*, the Modern Ukrainian *bib* and the Modern Serbian *bob* (42). The only descendant of Proto-Indo-European root-word where the meaning shifted was Old Greek, where as *φακóς* began and continued to denote ‘lentil’. The Celtic languages borrowed their words denoting field bean either from Latin, such in their Brythonic branch with the Modern Breton *fav*, or from the Germanic languages, such in the Goidelic branch with the Modern Irish *pónaire* (43). Similarly, the Slavic words were borrowed by neighbouring Indo-European languages, such as *boba* in the case of Romani and *bob* in the case of Romanian (44).

**Table 1:** Words denoting lentil, pea and field bean in the modern Indo-European languages of Europe.

Branch	Language	Lentil	Pea	Field bean
Albanian		*thjerrëz*	*bizele*	*bathë*
Armenian		*osp*	*olor*	*lobi*
Baltic	Latvian	*lēca*	*zirņi*	*pupas*
	Lithuanian	*lęšis*	*žirnis*	*pupa*
Celtic	Breton	*pizenn rous*	*piz*	*fav*
	Cornish		*pýsen*	*fav*
	Irish	*lintile*	*pis*	*pónaire*
	Manx	*pishyr lughag*	*pishyr*	*poanrey*
	Scottish Gaelic	*leantail*	*peasair*	*pònair*
	Welsh	*corbysen*	*pysen*	*ffa*
Germanic	Danish	*linse*	*ært*	*bønne*
	Dutch	*linze*	*erwt*	*boon*
	English	*lentil*	*pea*	*bean*
	Faroese		*ertur*	*bøna*
	Flemish	*lins*	*erwt*	
	Frisian		*eart*	*beanne*
	German	*Linse*	*Erbse*	*Bohne*
	Icelandic	*linsa*	*erta*	*baun*
	Norwegian	*linse*	*ert*	*bønne*
	Swedish	*lins*	*ärt*	*böna*
	Yiddish		*arbes*	*bob*
Hellenic	Greek	*fakí*	*bizéli*	*koukiá*
Indo-Iranian	Kurdish	*nîsk*	*polik*	
	Ossetic	*qædur*	*tymbylqædur*	*qædur*
	Romani		*boobi*	*boba*
Italic	Aragonese		*bisalto*	*faba*
	Aromanian		*grãshac*	
	Asturian		*arbeyu*	*faba*
	Catalan	*llentia*	*pèsol*	*fava*
	Corsican	*lentichja*	*pisu*	*fava*
	French	*lentille*	*pois*	*fève; fèverole*
	Friulian	*lint*	*bîsi*	*fave*
	Galician	*lentella*	*ervella*	*faba*
	Italian	*lenticchia*	*pisello*	*fava*
	Leonese	*llenteyas*	*arbeyu*	*faba*
	Ligurian	*lentìggia*	*poéixo*	*bazann-a*
	Occitan	*mendilh*	*pòis*	*fava*
	Picard		*pos*	*fèfe*
	Portuguese	*lentilha*	*ervilha*	*fava*
	Romanian	*linte*	*mazăre*	*bob*
	Romansh	*lentiglia*	*arveglia*	*fav*
	Sardinian	*lentígia*	*pisu*	*fa*
	Spanish	*lenteja*	*guisante*	*haba*
	Walloon	*lintile*	*peû*	*féve*
Slavic	Belarusian	*sačavica*	*garoh*	*bob*
	Bulgarian	*leshta*	*grah*	*bob*
	Croatian	*leća*	*grašak*	*bob*
	Czech	*čоčka*	*hrách*	*bob*
	Kashubian		*groch*	*bób*
	Lower Sorbian	*sok*	*groch*	*bob*
	Macedonian	*lekja*	*grašok*	*bob*
	Polish	*soczewica*	*groch*	*bób*
	Russian	*chechevitsa*	*gorokh*	*bob*
	Rusyn	*lenča*	*hraščok*	*bob*
	Serbian	*sočivo; leća*	*grašak*	*bob*
	Slovak	*šošovica*	*hrach*	*bob*
	Slovenian	*leča*	*grah*	*bob*
	Ukrainian	*sochevitsia*	*gorokh*	*bib*
	Upper Sorbian	*sok*	*hroch*	*bob*

**Figure 3:**
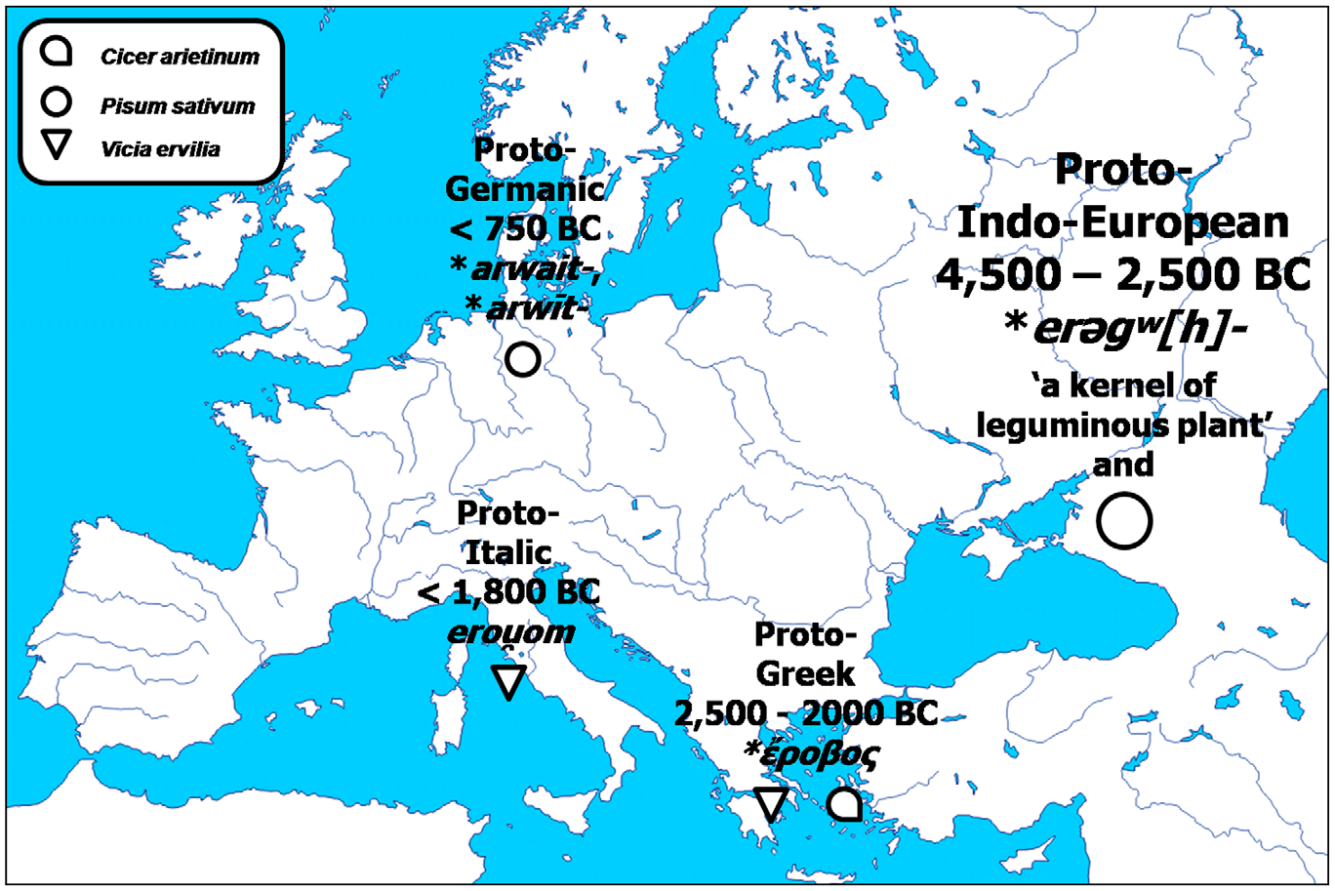
Initial evolution of the Proto-Indo-European root *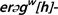.

Another Proto-Indo-European root-word with important derivatives (Fig. 3) is *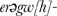, *eregw(h)o-*, *erogw(h)o-*, denoting both ‘the kernel of a leguminous plant’ and ‘pea’ (39, 40). The Proto-Germanic root-word *arwait-, *arwīt-, denoting ‘pea’, kept its meaning in its numerous descendants, such as the Modern Norwegian with *ert,* or the Standard Dutch with *erwt*, and the borrowings made during the great migrations of the Germanic tribes, found in several Italian dialects such as the West Lombard *erbion*. The Proto-Greek *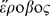 gave both the Old Greek *όροβος*, denoting ‘bitter vetch’, and *έρέβινθος*, denoting ‘chickpea’, with the latter evolving into Modern Greek *ρεβιθιά*, with the same meaning. The supposed Proto-Italic *erouom is judged to be a direct source of the well-known Latin *ervum*, denoting ‘bitter vetch’, from which, in turn, derive contemporary descendants denoting ‘pea’, such as the Portuguese *ervilha* (Table 1).

Of all its derivates, the Proto-Indo-European root-word *ghArs-, *ghers-2*, denoting ‘a leguminous plant’ (39, 40), was preserved in the Proto-Slavic *gorxŭ, with a shift of meaning to *pea* and producing modern forms denoting the same, such as the Czech *hrách*, the Russian *gorokh* and the Bulgarian *grah* (45).

**Figure 4:**
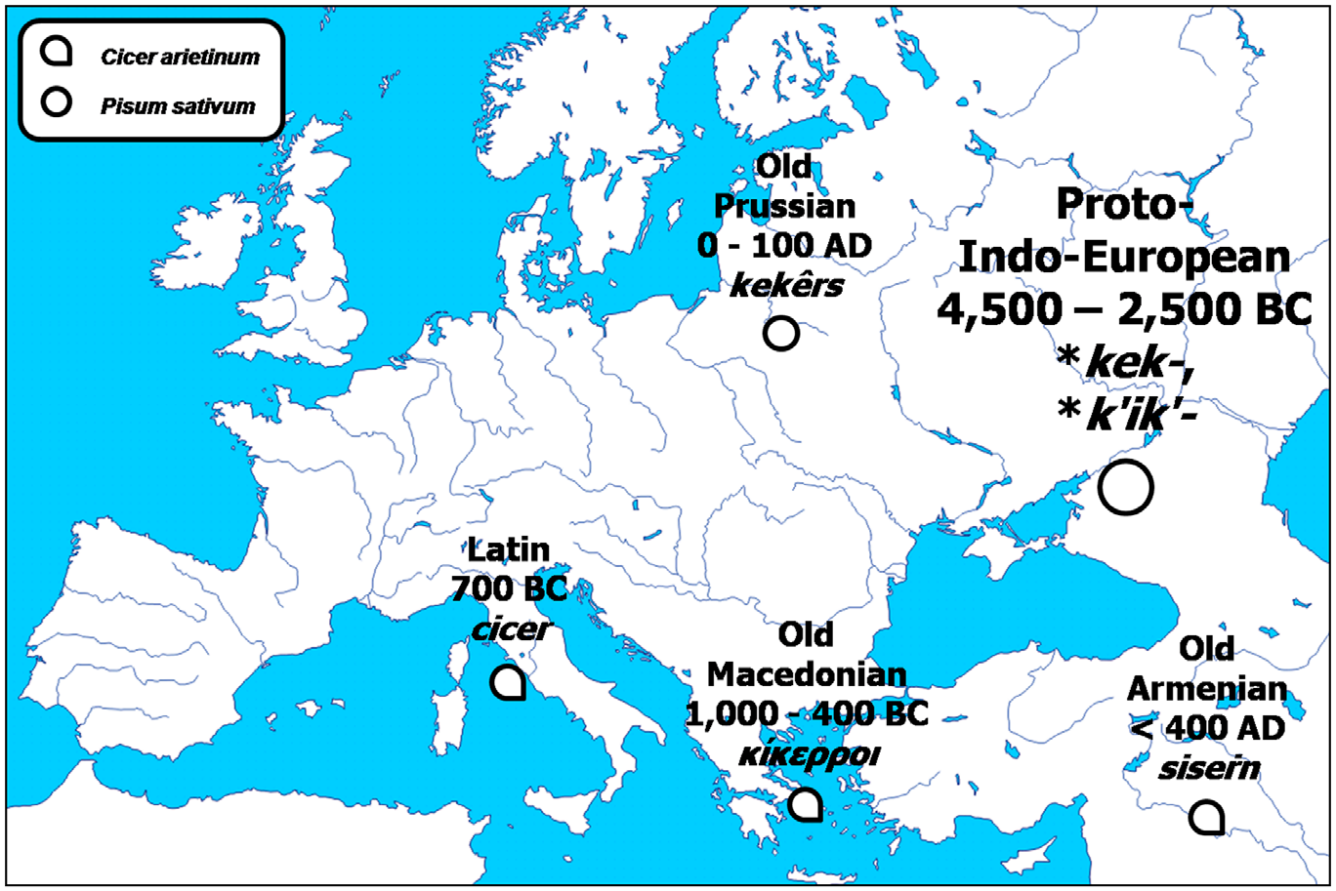
Initial evolution of the Proto-Indo-European root *kek-.

The original meaning of the Proto-Indo-European root-word *kek-, *k'ik'-, *kiker-*, namely *pea* (39, 40) was preserved only in the extinct Old Prussian language (Fig. 4). In all other attested derivatives, it began to denote ‘chickpea’. The Old Armenian *siseŕn* gave the Modern Armenian *siser* and the Latin *cicer* produced numerous descendants such as the Catalan *cigró* and the French *pois-chiche*, by way of a kind of pleonasm. The Old Macedonian *κίκερροι*, denoting ‘chickpea’ and possibly being derived from the Proto-Hellenic *κικριός, left no attested forms in its descendants.

**Figure 5:**
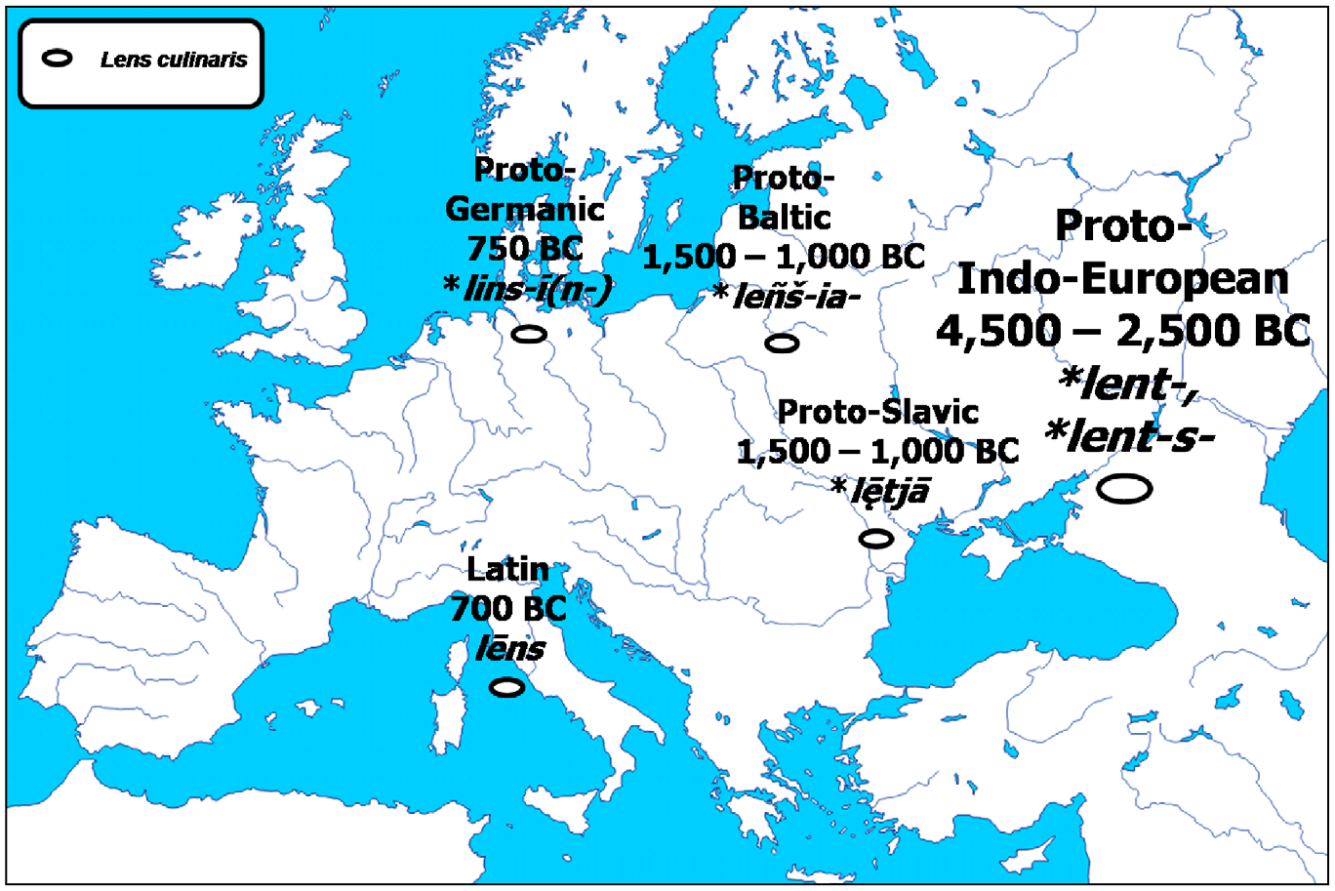
Initial evolution of the Proto-Indo-European root *lent-.

The Proto-Indo-European root-word *lent-, *lent-s-, denoting ‘lentil’ (39, 40), proved remarkably conservative in morphology and meaning, both among its direct derivatives (Fig. 5) and its modern descendants (Table 1). The Proto-Baltic * *-ia-* gave the Modern Latvian *lēca*; the Proto-Germanic *lins-ī(n-) gave the Modern Icelandic *linsa* and the Modern Swedish *lins*; the Latin *lēns* gave the Modern Corsican *lentichja* and the Modern Occitan *mendilh*; the Proto-Slavic *lētjā gave the Serbo-Croatian *leća* and Slovenian *leča* (46).

The attested Proto-Indo-European root-words directly linked to pulse crops are further testimony that Proto-Indo-European society was well-acquainted with agriculture (47), and was not predominantly nomadic and pastoral, as initially thought by the proposers of the Kurgan hypothesis (48). As already noted, the Proto-Indo-European root-word denoting ‘field bean’ had a primarily descriptive character. Such cases are widely present in linguistic development (49), and there are several more Proto-Indo-European root-words that originally had no direct link to pulses, but began to denote them in their derivatives. It’s worth mention that the Latin *legūmen*, denoting ‘pod’, evolved from the Proto-Indo-European *leg'-*, meaning *to gather*; that the Latin *pisum* was derived from the Proto-Indo-European *pis-, meaning *to thresh*; and that the Latin *vicia*, through its verb *vincīre*, meaning *to bind* and obviously referring to vetches’ tendrils, originated from the Proto-indo-European *weik, meaning *something pliable*, perhaps pointing at their slender and climbing stems (50).

### Uralic and Altaic Languages

The words denoting pulse crops in the modern Uralic languages of Europe that either have the most numerous speakers, and are the best studied, such as Estonian, Finnish or Hungarian, are mostly borrowings. The Finnic languages of the Uralic language family represent the westernmost spread of this family, comprising Finnish, Estonian, Karelian and several more languages also spoken in the Baltic region. The words denoting ‘pea’ in all these languages, such as the Estonian *hernes*, the Finnish *herne* or the Karelian *herneh* (Table 2), are the early borrowings from the neighboring Indo-European Baltic languages (22), witnessed by the Proto-Baltic root *žirn-ia-, *žirn-i˜ă, also denoting ‘pea’ (39, 40). However, the word denoting ‘pea’ in the Saami language, spoken in the utmost north of Scandinavia, namely *hearta*, could be a borrowing from Germanic languages. The words denoting ‘field bean’ in both Finnic and Saamic languages are largely borrowings from Slavic, such as the Estonian *uba*, the Finnish *papu* and the Saami *báhpu*. Magyar, having separated from its Finno-Ugric stock quite early (33), also borrowed some words from the Slavic tribes already living in Pannonia, with *bab* denoting ‘field bean’ and *lencse* denoting ‘lentil’.

**Table 2:** Words denoting lentil, pea and field bean in the modern Uralic languages of Europe.

Branch	Language	Lentil	Pea	Field bean
Finno-Permic	Erzya		*ksnav*	*kuvtjol*
	Estonian	*lääts*	*hernes*	*uba*
	Finnish	*linssi*	*herne*	*papu*
	Ingrian		*herne*	*papu*
	Karelian		*herneh*	*papu*
	Komi		*an’kytsh*	*pubād*
	Livonian		*jernõd*	* 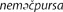 *
	Moksha	*babanjsnavna*	*snavnja*	*babanjsnav*
	Saami		*earta; hearta*	*báhpu*
	Udmurt	*jasnyk*	*köžy*	*s’öd köžy*
	Veps		*herneh*	
	Võro	*lääts*	*herneh*	*uba*
Ugric	Hungarian	*lencse*	*borsó*	*bab*
	Khanty		* 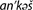 *	
	Mansi		*an’kas*	

**Figure 6:**
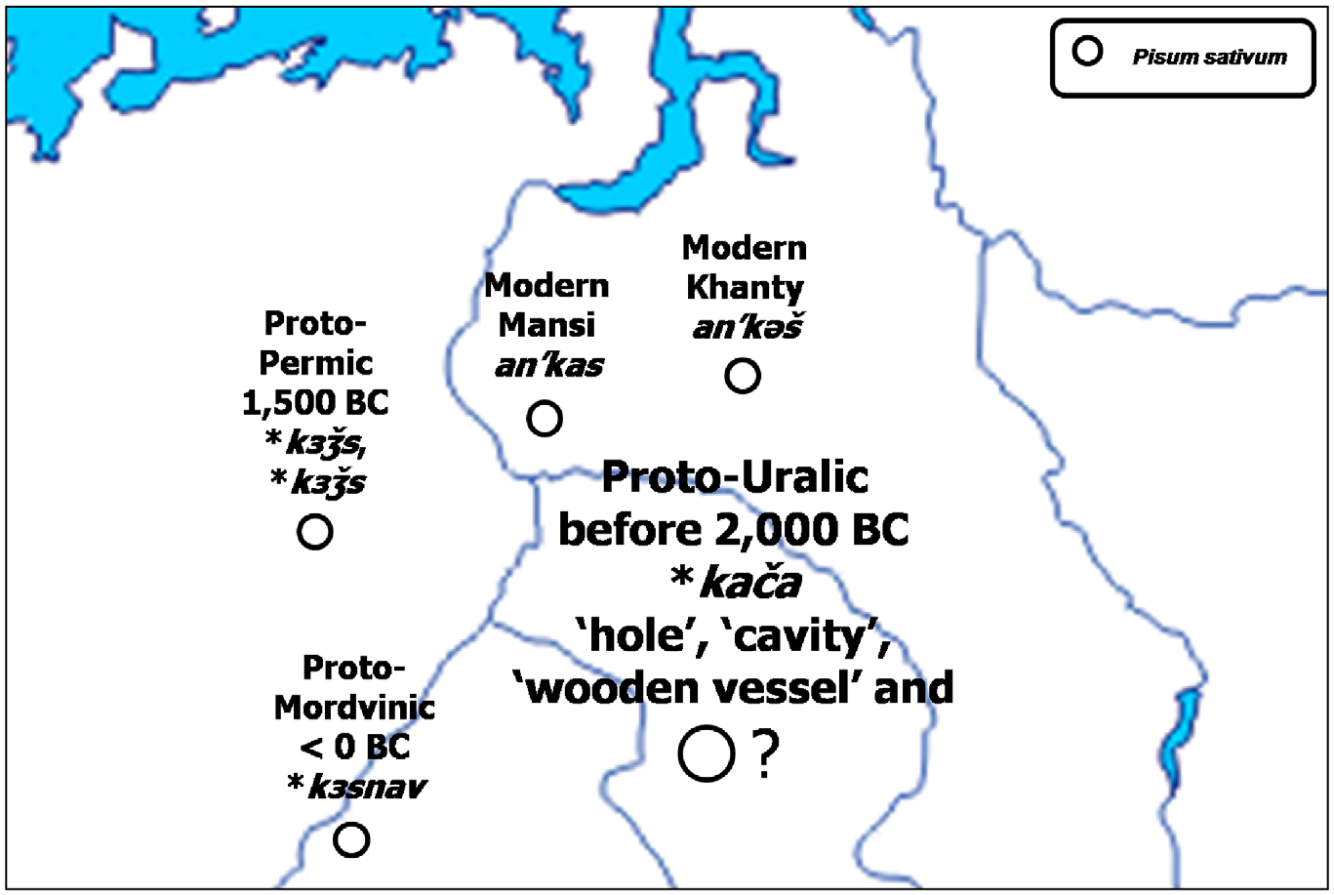
Supposed evolution of the Proto-Uralic root *kača.

However, in case of the Finno-Ugric languages still spoken in areas close to the supposed Proto-Uralic homeland, such as Permic and Mordvinic, there exists a great morphological similarity in their words denoting ‘pea’ (Fig. 6). The Proto-Permic *kзžs, giving the Modern Komi *an’kytsh* with and the Modern Udmurt *köžy*, fully corresponds to the Proto-Mordvinic *kзsnav, evolving into the Modern Erzya *ksnav* and the Modern Moksha *snavnja*, all denoting ‘pea’ (51). They are also equivalent to the words denoting ‘pea’ in Khanty and Mansi, the closest relatives of Magyar, with 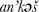 in the former and *an’kas* in the latter. There is a Proto-Uralic root-word that could be a candidate for the still unattested ancestral form denoting ‘pea’ in this language family: the Proto-Uralic *kača denoted ‘hole, cavity’ and ‘a wooden vessel’ (52), and the possibility that it also described the act of hollowing the pea seeds out of their pods, or the vessel-like form of a pea pod, still remains to be assessed by a detailed linguistic analysis.

**Figure 7:**
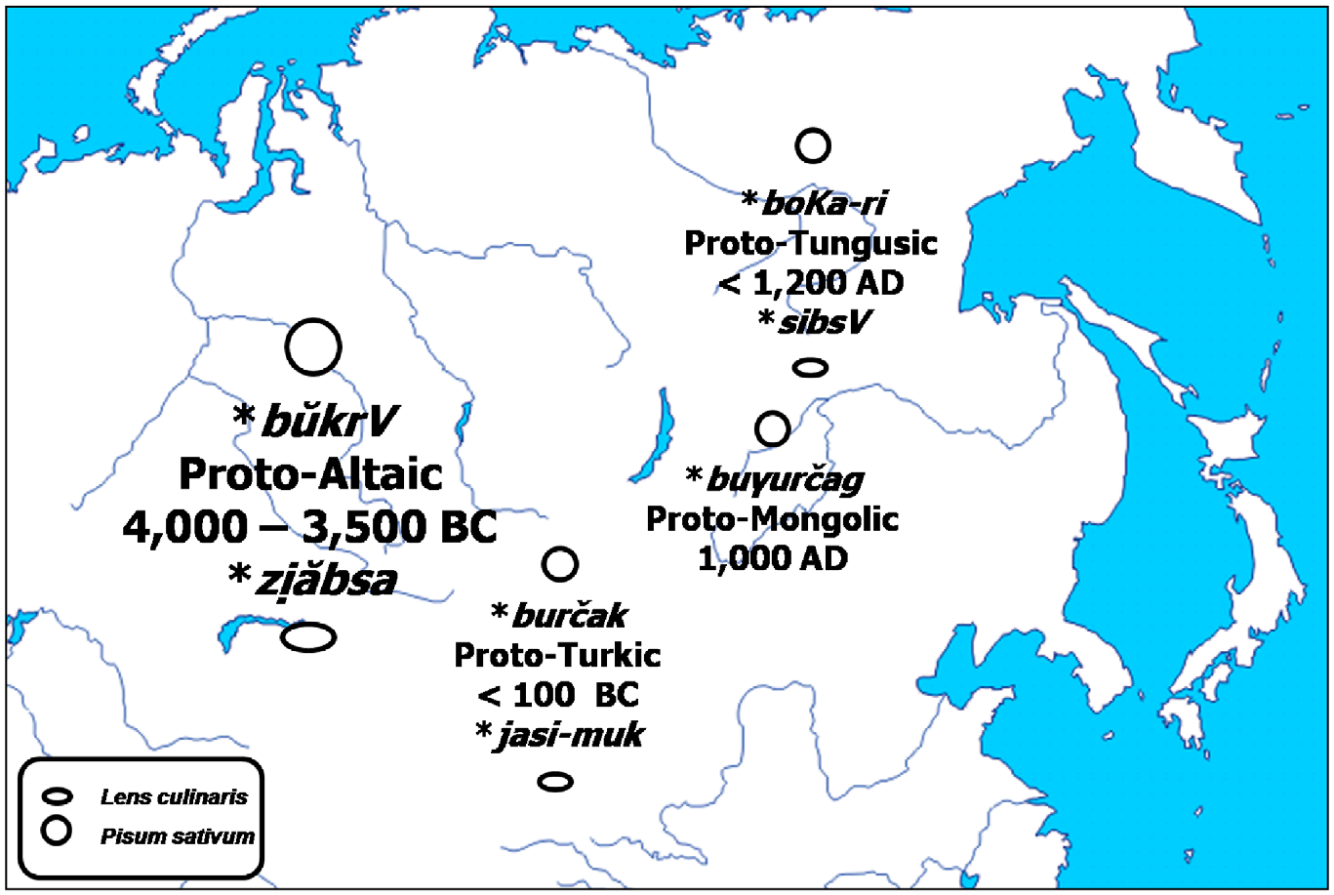
Initial evolution of the Proto-Altaic roots *bŭkrV and *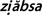.

One of the two attested Proto-Altaic root-words related to pulses is *bŭkrV (Fig. 7), denoting ‘pea, nut’ and ‘cone’ (53). Its descendants with unchanged meaning are the Proto-Turkic *burčak, the origin of all the words denoting ‘pea’ in the majority of the modern Turkic languages of Europe (Table 3), such as *burşaq* in Kazakh or *borchaq* in Tatar, and the Proto-Mongolic *buγurčag, giving the Modern Kalmyk *bürcëg*. In the early days of separating from their Ugric relatives and at the outset of their great migration towards their present home in Central Europe, the Magyar-speaking Uralic tribes borrowed the word denoting ‘pea’ from their Turkic neighbours, and adopted it as *borsó* (54). Another Proto-Altaic root, *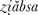, denoted primarily ‘lentil’ (53) and gave the root-words denoting the same in the Proto-Turkic, *jasi-muk, and the Proto-Tungusic, *sibsV (Fig. 7). From the Turkic, the word denoting ‘lentil’ was borrowed by neighbouring languages belonging to other families, such as the Uralic Udmurt with *jasnyk* (46).

**Table 3:** Words denoting lentil, pea and field bean in the modern Altaic languages of Europe.

Branch	Language	Lentil	Pea	Field bean
Mongolic	Kalmyk	*nyet ulan burtsg*	*bürcëg*	*bob*
Turkic	Azeri	* 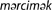 *	*noxud*	*lobya*
	Bashkir	*jasmyq*	*borsaq*	*baqsa borsağı*
	Chuvash		*pärça*	*nímëş parşí*
	Crimean Tatar	*bercimek*		*pasle*
	Gagauz	*mercimek*	*borchaq*	
	Karachay-Balkar		*burchaq*	*hans qudoru*
	Karaim		*burchax*	*bob; burcacyk*
	Kazakh	*jasimiq*	*noqat; burşaq*	*iri burşaq*
	Kumyk		*burchaq*	*burçaq*
	Nogai		*burşaq*	
	Tatar	*jasmyq*	*borchaq*	*bakça borçagı*
	Turkish	*mercimek*	*bezelye*	*bakla*

### Caucasian, Basque and Other European Languages

The Proto-Caucasian root-word *hōwł(ā) denoted both ‘bean’ and ‘lentil’ (55), and gave the words denoting either one or the other crop in its modern descendants within the Avar-Andi-Dido group, such as *holó* in Avar, denoting ‘field bean’, and *hil* in Tsez, denoting ‘pea’ (Table 4). Another Proto-Caucasian root-word, *qŭr’ā, denoting exclusively ‘pea’ (55), gave rise to words of the same meaning in most other languages of the Daghestani group, such as the Lak *quIru* or the Lezgi z*ar*, as well as in the languages of the Abkhazo-Adyghean group, such as the Kabardian *cesh*. Interestingly enough, it is within the languages of the Nakh group where the meaning shifted from *pea* to *field bean*, such as in Chechen *qö* and Ingush *qe*, with a possible borrowing into Adyghe, also known as Circassian (in a narrow sense), with *ceshä*, and the Indo-Iranian Ossetic, with *qædur* (44).

**Table 4:** Words denoting lentil, pea and field bean in the modern Caucasian languages.

Branch	Sub-branch	Language	Pea	Field bean
Northeast (Nakh-Daghestanian)	Avar-Andi-Dido	Andi		*holi*
		Akhvakh		*hali*
		Avar		* 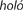 *
		Bagvalal	*hal*	
		Bezhta		*holo*
		Botlikh	*hali*	
		Chadakolob	* 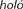 *	
		Chamalal		*hal*
		Godoberi	*hali*	
		Hinukh	*hilu*	
		Hunzib	*helu*	
		Inkhokvari	*hel*	
		Karata		*hale*
		Khwarshi		*ħel*
		Tindi		*hali*
		Tsez	*hil*	
	Lak-Dargwa	Akusha	*qara*	
		Chiragh	*qara*	
		Dargi	*qara*	
		Lak	*quIru*	*luħi qjuru*
	Lezgic	Aghul	*xur*	
		Archi	*čaq*	*bex:`? čaq*
		Kryts	*xarxar*	
		Lezgi	*nahut; zar*	*xaru; paxla*
		Rutul	*xar*	
		Tabasaran	*harar; xar*	*xaru*
		Tsakhur	*xara*	
	Nakh	Chechen	*qöş*	*qö*
		Ingush	*gerga qeŝ*	*qe*
Northwest (Abkhazo-Adyghean)	Circassian	Abaza	*k’yrk’yrlaš*	
		Abkhaz	*k’yrk’yrra*	
		Adyghe	*nekhut*	*ceshä*
		Kabardian	*cesh*	

**Figure 8:**
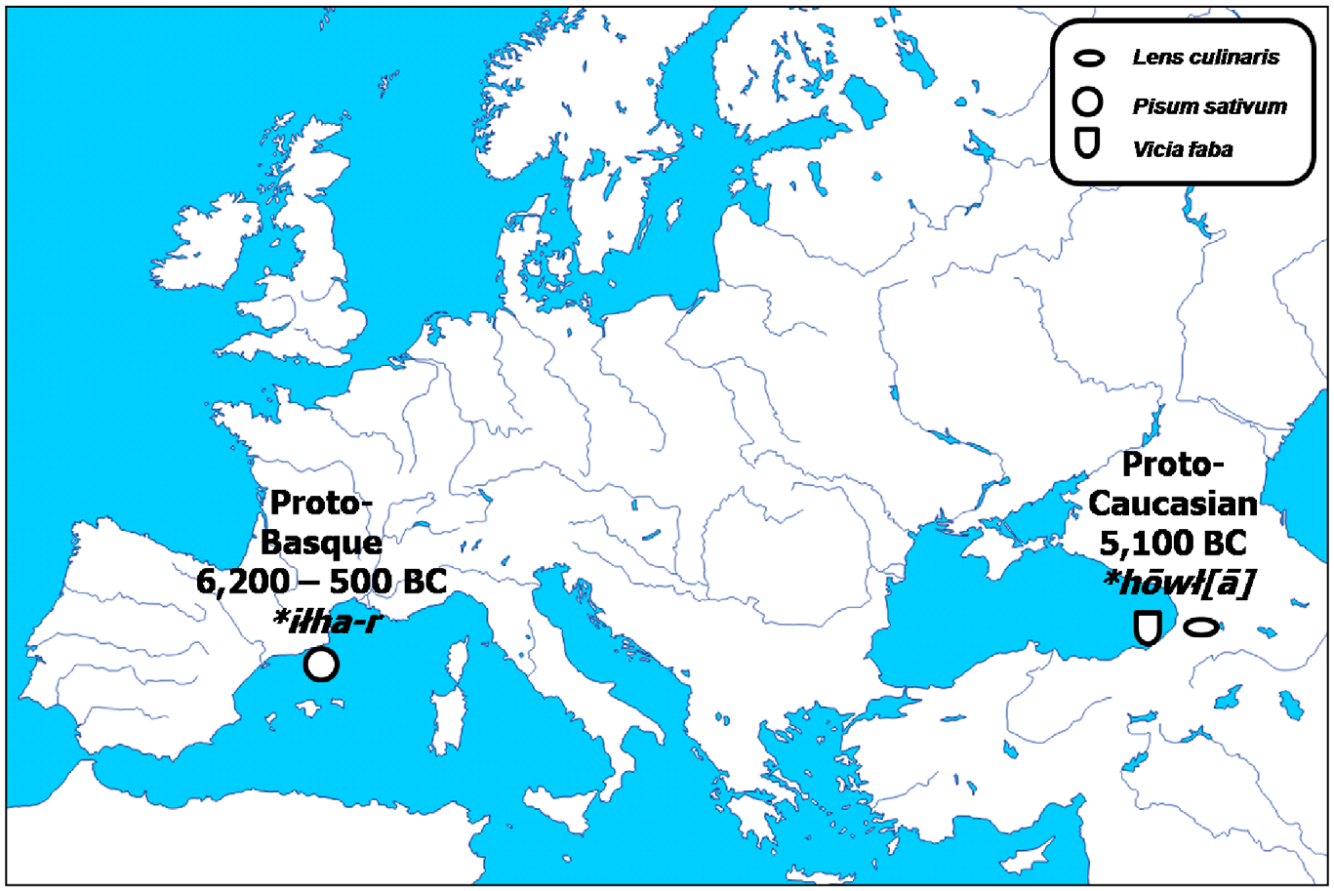
Derivation of the supposed Proto-Sino-Caucasian root *hVwłV in its Caucasian and Basque descendants.

According to the hypothesis concerning the existence of the Dené-Caucasian language superfamily, the Caucasian languages are related to Basque and several other language isolates in Asia and North America. Genetic studies have already provided some evidence to this effect, suggesting that both the Basque and the North Caucasian peoples could be the descendants of Palaeolithic hunter-gatherers that retreated into the mountains when the last Ice Age ended, and new peoples began to inhabit Europe (56). The said Proto-Caucasian root-word *hōwł(ā) is thus brought into connection with the Proto-Basque root-word *iłha-r (Fig. 8). Originally, the latter denoted ‘pea, faba bean, vetch’ and ‘heather’ (57), but survived into the modern times as the Basque *ilar*, denoting exclusively ‘pea’. The supposed common ancestor of both the Proto-Caucasian and the Proto-Basque stocks is the Proto-Sino-Caucasian *hVwłV, ultimately denoting ‘field bean’ (58).

The research on the words denoting pulses in the Kartvelian languages did not result in any attested Proto-Kartvelian root-words. If they ever existed, it is most likely that they were gradually replaced by the borrowings from diverse neighboring languages, such as the Indo-Iranian Persian, in the case of the Georgian *mukhudo*, Altaic, in the case of the Laz *parsuli*, and Caucasian, in the case of the Svan *ghedar*, all denoting ‘pea’ (45).

The Maltese language preserved the memory of two Proto-Semitic root-words related to pulses. One of them is *'adaš-, denoting ‘lentil’ (59), with the Modern Maltese form of *ghads*, denoting the same. Another one, *pūl-, denoted ‘field bean’, and as *fula* was preserved in Modern Maltese together with its original meaning. It could also be responsible for the Indo-Iranian Kurdish word denoting ‘pea’, *polik*.

With great certainty and on the basis of the presented etymological evidence, it may be claimed that the most ancient Eurasian pulse crops, such as the pea, the lentil and the field bean, were surely among the basic components of Proto-Indo-European farming systems. It is also notable that the frequently migrating and complexly evolving Indo-European peoples preserved the words denoting these crops that they used in their original homeland, and continued to use them in their new territories, often loaning them to both aboriginal populations and to those that came afterwards.

It may be assumed that the pea played the most prominent role of all the pulses among the ancient Uralic tribes, since, judging by their morphology only, the words denoting ‘lentil’ and ‘field bean’ in these languages are usually based upon the words denoting ‘pea’. Similarly, it seems that peas and lentils were the dominant pulse crops grown by the ancestors of the modern Altaic nations, since their words denoting ‘field bean’ and other grain legumes are based either upon the words denoting ‘pea’ or ‘lentil’, or are mostly borrowings from Persian. On the other hand, peas and field beans played the most important role among the Caucasian peoples, since the number of attested words related to ‘lentil’ was extremely small, and largely based upon those denoting ‘pea’ or ‘field bean’.

The presented results prove that the most ancient Eurasian pulse crops, especially the pea, the lentil and the field bean, were well-known and most likely extensively cultivated by the ancestors of all modern European nations, regardless of exactly where in Europe or Asia their proto-languages developed and began to diversify. In most cases, the root-words of these proto-languages proved to be remarkably well-preserved in both morphology and meaning, though there is a rich testimony of considerable and lively contact between different language families, and of extensive mutual exchange of the words denoting pulses. The attested lexicological continuum witnesses the existence of millennia-long links between the peoples of Eurasia to mutual benefit, and hopefully encourages the much closer collaboration of all those dealing with the agricultural history of the Old World.

## Materials and Methods

In order to carry out the practical side of its first goal, and thus establish the fundamentals of achieving the second one, this preliminary research was aimed at a detailed search of all available printed and electronic resources related to the etymology of the languages spoken in Europe for root-words related to pulse crops and leguminous plants in general. Numerous printed and electronic dictionaries of modern European languages were used as an auxiliary tool, by compiling the words denoting ‘pea’, ‘lentil’, ‘field bean’ and other traditional and most ancient Eurasian pulse crops. The whole outcome of this lexicological screening of modern European languages is not presented in this short communication, as its sheer magnitude demands completely separate processing and presentation. It was used simply as a guide to, and confirmation of, the said etymological research. Each of the present language families of Europe was dealt with individually, and the results are presented accordingly. Where more than one was assessed, the root-words were listed in alphabetical order. The attested borrowings of words derived from these root-words, whether between languages belonging to different branches of the same family, or between languages of different families, were also recorded.
